# Supplementary material for: Complexome profiling of the Chlamydomonas psb28 mutant reveals TEF5 as an early PSII assembly factor
Source: Plant Cell. 2025 Mar 17;37(6):koaf055. doi: 10.1093/plcell/koaf055 (PMC12164590; doi:10.1093/plcell/koaf055)
Supplement: koaf055_Supplementary_Data [file koaf055_supplementary_data.zip › psb28_interactive.zip!/plots/intensity/qualityPlots/peptides/GCST_Cre03.g193750.t1.1.html]

### Cre03.g193750.t1.1

Trivial name: GCST  
SeqCoverage [median]: 34.40  
Mass: 44.63 kDa  
  
 

| Sequence | MissedCleavage | Mass [Da] | wtIntensity | muIntensity | Score |
| --- | --- | --- | --- | --- | --- |
| DGTGSLSAFTNEK | 0 | 1325.61 | 1309.08 | 4092.34 | 177.44 |
| EKFDFLGGDIIKK | 2 | 1508.82 | 998.06 | 0.00 | 54.34 |
| FDFLGGDIIKK | 1 | 1251.69 | 1052.47 | 571.34 | 48.28 |
| GGIIDDTVITK | 0 | 1130.62 | 3510.11 | 5028.41 | 64.71 |
| GKVNDAVVTK | 1 | 1029.58 | 1022.92 | 1448.72 | 101.65 |
| GLDVAMTVHDDR | 0 | 1327.62 | 5294.19 | 8539.65 | 81.49 |
| MAGLGPR | 0 | 700.37 | 0.00 | 1033.41 | 91.09 |
| MPFVPTPYYK | 0 | 1241.62 | 3545.45 | 7730.20 | 111.04 |
| NIAMGYVDKDFAK | 1 | 1470.72 | 617.00 | 1401.90 | 51.22 |
| QHSVVSTPDGK | 0 | 1153.57 | 561.89 | 0.00 | 74.39 |
| QLAEGVSK | 0 | 830.45 | 617.33 | 0.00 | 91.06 |
| RVGFVSTGAPAR | 1 | 1216.67 | 320.51 | 683.78 | 54.34 |
| TGYTGEDGFEISVPNSHAVALAEK | 0 | 2491.19 | 2673.61 | 4916.00 | 88.87 |
| VGFVSTGAPAR | 0 | 1060.57 | 12077.57 | 6881.23 | 102.07 |
| VNDAVVTK | 0 | 844.47 | 0.00 | 1320.96 | 81.30 |
|  |  |  | 33600.2 | 43647.9 |  |
